# Generation of picosecond pulses using soliton compression in a dual cavity laser

**DOI:** 10.1038/s41598-025-07313-x

**Published:** 2025-07-01

**Authors:** Martin Brunzell, Max Widarsson, Fredrik Laurell, Valdas Pasiskevicius

**Affiliations:** Department of Applied Physics, KTH Royal Institute of Technology, Albanovägen 29, Stockholm, 114 19 Sweden

**Keywords:** Solid-state lasers, Nonlinear optics

## Abstract

In this work, the development of a novel ultra-short laser system is presented, building upon previous research in passive mode-locking using cross-amplitude modulation (XAM). By combining XAM with cascaded second-order nonlinearity mode-locking (CSM) the system produced a stable bright-dark two-color output with picosecond pulses and a repetition rate of 275 MHz with an average output power of 100 mW for an 808 nm pump power of 4 W. The experimental setup involved two Nd: YVO_4_ lasers operating at 1064 nm and 1342 nm, where the two cavities were interconnected with a dichroic mirror allowing for a shared section where a periodically poled KTiOPO_4_ (PPKTP) was introduced. In the separate sections, the independently diode-pumped laser crystals were placed. The enhanced intra-cavity intensity achieved through XAM enabled effective pulse compression via CSM. The results demonstrate the system’s ability to generate near-transform-limited pulses as short as 14 ps, offering potential for applications such as medical imaging and LIDAR.

## Introduction

Ultra-short laser systems have demonstrated immense utility across a diverse set of applications, such as biological imaging^1^, high-precision machining^2^, and nonlinear spectroscopy^3^. Currently, most ultrashort pulse laser systems rely on passive mechanisms such as Kerr lens mode-locking (KLM), that depends on a $\:{\chi\:}^{\left(3\right)}$ nonlinearity. This can be reliably implemented in the femtosecond regime, where the high peak powers can compensate for the low values of $\:{\chi\:}^{\left(3\right)}$ found for most solid-state materials^4^. However, for many applications such as minimally invasive surgery^5^, material processing^6^, and light detection and ranging (LIDAR)^7^ longer pulses can be beneficial. Another mode-locking technique that is commonly implemented in the picosecond regime is cascaded second-order nonlinearity mode-locking (CSM) where a nonlinear crystal imprints a nonlinear phase shift, thereby creating a large, effective $\:{\chi\:}^{\left(3\right)}$ nonlinearity^8^. This is possible through a cascaded degenerate $\:{\chi\:}^{\left(2\right)}:{\chi\:}^{\left(2\right)}$ interaction of second harmonic generation (SHG) and back-conversion. It enables the nonlinear crystal to act as an intensity-dependent lensing element, just as in the conventional KLM case. The difference is that the process is based on cascaded second-order nonlinearities, resulting in a larger nonlinearity suitable for lower peak powers found in the picosecond regime and high repetition rates^9^. The nonlinear medium is detuned from perfect phase matching for SHG, and by selecting the appropriate phase mismatch, the sign and magnitude of the effective $\:{\chi\:}^{\left(3\right)}\:$can be engineered. Sources using CSM have demonstrated pulses as short as 2.8 ps in Nd: GdVO_4_ at 1063 nm^10^, 4.8 ps in Nd: BaY_2_F_8_ at 1049 nm^11^ and 3.6 ps in Nd: YVO_4_ at 1340 nm^12^. A related cascaded $\:{\chi\:}^{\left(2\right)}:{\chi\:}^{\left(2\right)}$interaction resulting in unbalanced type-II SHG has been exploited for intensity-dependent polarization evolution and self-starting mode-locking in continuous wave (CW) lasers^13^. The modulation depth and bandwidth with CSM is primarily influenced by cavity design^14^. Additionally, the absence of SHG buildup enhances laser stability. Importantly, CSM relies on the effective Kerr effect due to the phase-mismatched second-order interaction, which is not limited by the SHG acceptance bandwidth. Therefore, pulse compression into the fs pulse range and effective soliton regimes can be realized^15–17^.

In our previous work^18^, we introduced a novel passive mode-locking mechanism using cross-amplitude modulation (XAM) in the common arm of two laser cavities. By precisely matching the cavity lengths, we generated two-color synchronized bright-dark pulse trains, with the bright pulses having a duration of approximately 250 ps. The mode-locking in such a cavity is based on the system’s self-organization to the oscillation state where sum-frequency mixing (SFM) losses are minimized. In this work, we show that once XAM establishes this self-organized synchronous bright-dark pulse oscillation, we can exploit phase-mismatched SHG in the same intracavity nonlinear crystal to achieve spectral broadening and compress the bright pulse train by about 18 times. The effective Kerr coefficient due to the cascaded $\:{\chi\:}^{\left(2\right)}:{\chi\:}^{\left(2\right)}$ interaction is negative, which promotes intracavity soliton compression under normal dispersion conditions at 1064 nm. The associated defocusing Kerr lens also promotes lower-loss fundamental mode oscillation, making the short pulse oscillation regime preferential. The mechanism allows for a stable mode-locked generation of picosecond pulses.

## Experimental setup

**Fig. 1:**
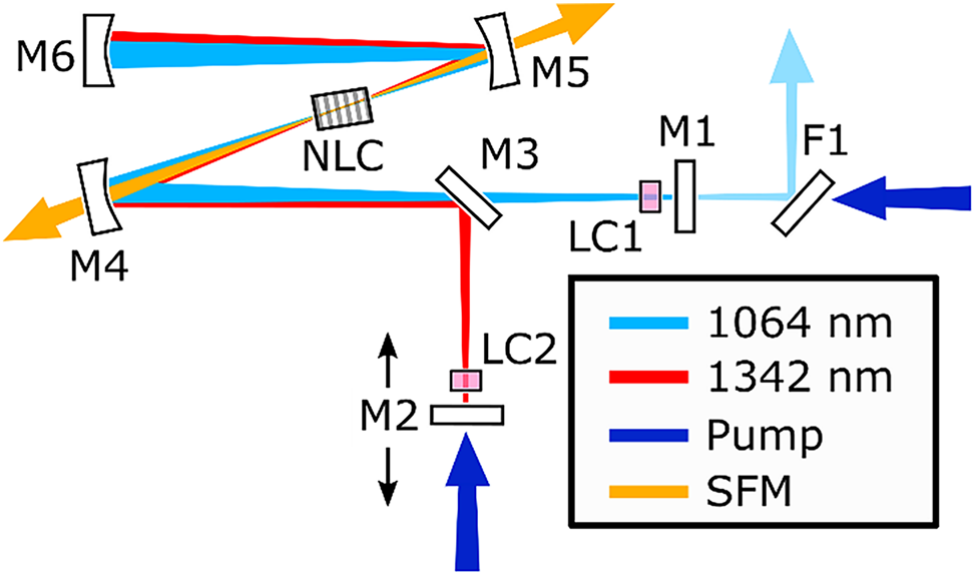
Experimental setup of the folded y-cavity. M1 acts as an output coupler for the 1064 nm arm with a reflectivity of 99.4%. M2 is HR at 1342 nm and HT for the pump at 808 nm. M3 is a dichroic mirror allowing the separation of the two arms with HT 1064 nm and HR for 1342. M4, M5, and M6 are HR 1064 and 1342 nm with the respective radius of curvature: 150, 100, and 150 mm. The dark blue arrows show the pumping.

The setup is illustrated in Fig. 1. The system consists of two diode-pumped (at 808 nm) Nd: YVO_4_ lasers operating at 1064 nm and 1342 nm, respectively. The design features a folded y-cavity, where two Nd: YVO_4_ lasers, operating at different wavelengths, are interconnected through a dichroic mirror. The cavity has a shared section where both lasers overlap and two separate paths where each Nd: YVO_4_ crystal (LC1,LC2), with appropriate AR coating, is located. The Nd: YVO_4_ crystals are cut along the a-axis with dimension 3 × 3 × 4 mm^3^ and a Nd doping of 1 at%. A periodically poled KTiOPO_4_ (PPKTP) crystal in the shared section enables phase-matched SFM between the two wavelengths. The laser operating at 1064 nm is comprised of the mirrors M1 and M4-M6. M4 to M6 are both high reflective (HR)-coated (*R* = 99.95%) for 1064 nm and 1342 nm, while M1 acts as an output coupler with 99.4% reflectivity at 1064 nm. A low-transmission output coupler was selected to enhance intra-cavity peak power and maximize the efficiency of the cascaded nonlinear interaction. The dichroic filter F1 transmits the pump and reflects the output at 1064 nm. The 1342 nm cavity consists of the mirrors M2, M3, and M4-M6, where the dichroic mirror M3 aids in promoting lasing at 1342 nm. Mirror M3 is HR (*R* = 99.5%) at 1342 nm and HT (T = 99.97%) at 1064 nm and combines/separates the two beams. Mirror M2 is HR for 1342 nm and HT for 808 nm. The 10 mm long PPKTP sample has an 8 mm long grating and a period of 12.65 μm that phase-matched SFM between 1064 nm and 1342 nm. A translation stage on the M2 mirror (see Fig. 1.) allows fine-tuning of the cavity length, addressing any mismatch in the two cavities’ roundtrip time and ensuring that they could be equally matched. This mode-locking is self-starting when the roundtrip times of both cavities are matched.

## Results and discussion

The different emission cross-sections for 1064 nm and 1342 nm transitions mandate different pump powers for the two lasers for self-starting mode-locking operation. In the following results, a pump power of 4 W and 7.5 W was used for the 1064 nm and 1342 nm pumps, respectively. The pump for 1064 nm was limited to 4 W, limiting the mode-locked output power. The power scaling is limited to damage threshold in the nonlinear medium and mirrors which can be increased by changing the cavity conditions for larger spot sizes. This limitation is not intrinsic to the technique itself but arises from the available pump diode power in the current setup. To scale up the power, the cavity design would need to accommodate for increased thermal lensing in the gain media as well as in the nonlinear crystal.

**Fig. 2:**
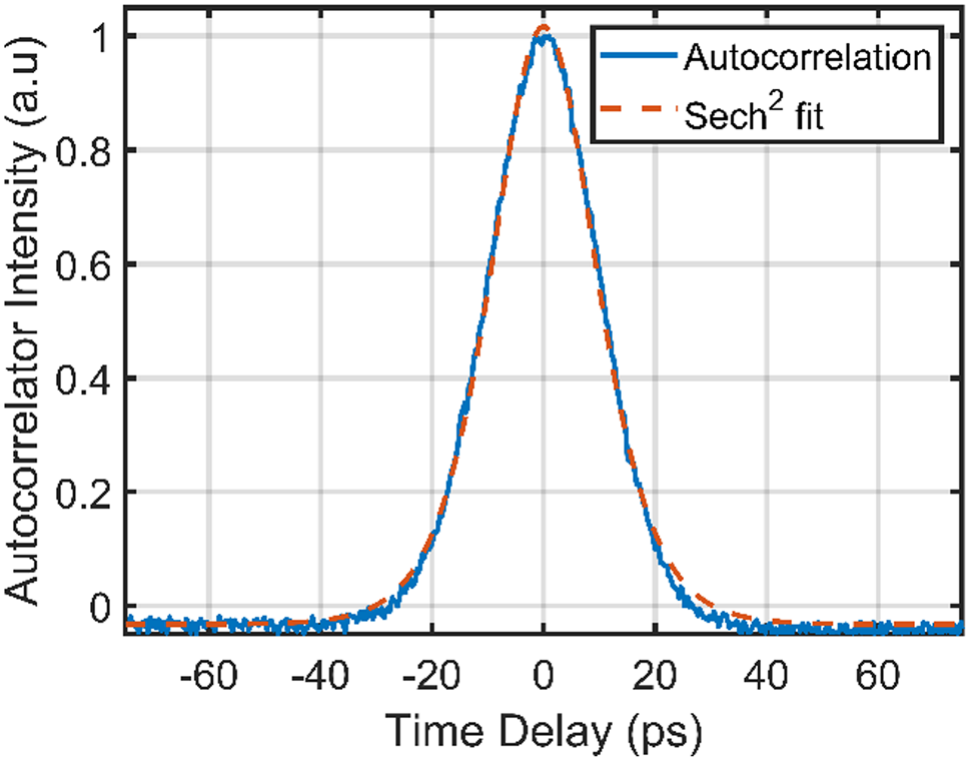
The autocorrelation trace for the 1064 nm bright pulses is shown. A FWHM of 22 ps is observed, which corresponds to a pulse width of approximately 14 ps for a sech²-shaped pulse.

An output power of 100 mW was obtained for the bright pulse train through M1. All mirrors in the 1342 nm cavity had high reflectivity for that wavelength. However, 20 mW of power that leaked out through the HR mirrors was used for pulse characterization. The reflectivity of 99.95% for the HR mirrors means that the intra-cavity power is 40 W for the 1342 nm laser. The pulse characterizations of the bright pulses were done using autocorrelation, where the resulting trace is shown in Fig. 2. The sech^2^-shaped pulses had a pulse width of ~ 14 ps (autocorrelation width of ~ 22 ps) and a repetition rate of 275 MHz. This corresponds to an intra-cavity pulse energy of 61 nJ, with a peak power of 4.34 kW and an intra-cavity average power of 16.7 W.

**Fig. 3:**
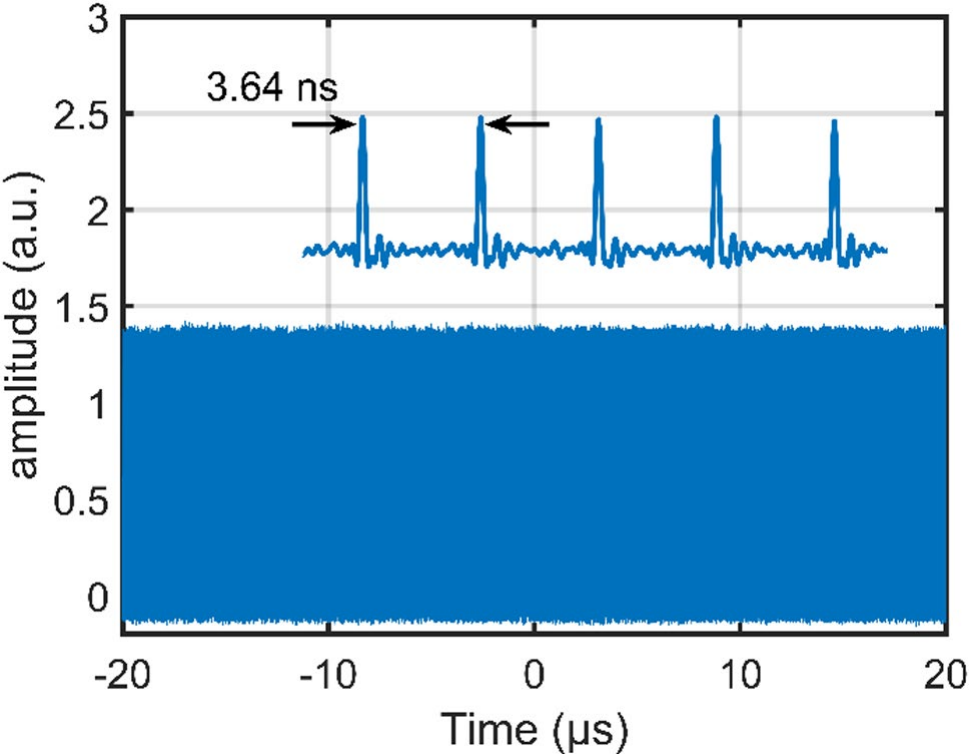
Power stability measurement throughout 40 µs is demonstrated with a peak power fluctuation of less than 1.4%. The inset above shows a zoomed-in picture of the trace (not to scale) with a period of 3.64 ns, corresponding to 275 MHz repetition rate.

**Fig. 4:**
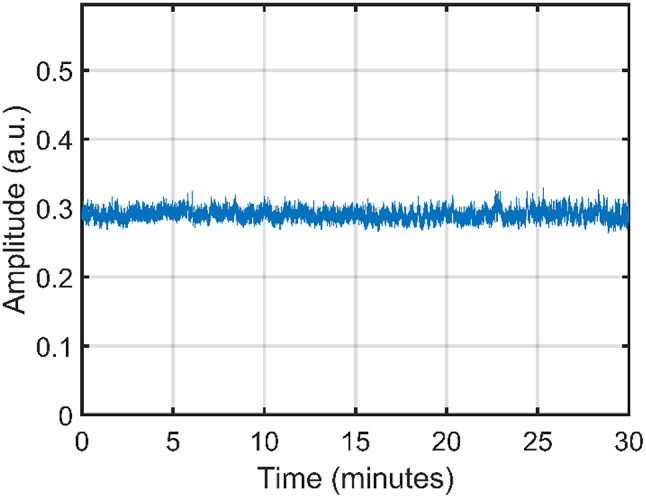
Stability measurement over 30 min, demonstrating a power fluctuation of less than 2.5%.

Power stability was studied under both short- and long-term conditions. Figure 3 shows power measurement over 40 µs as measured with a fast photodetector and displayed on an oscilloscope, with a resulting peak power fluctuation of less than 1.4%. Since this time scale is in the same order of magnitude as the fluorescence lifetime observed in Nd: YVO_4_, it shows that the mode-locking mechanism is inherently stable. A 30-minute stability test is presented in Fig. 4, demonstrating power fluctuations of less than 2.5%. The laser operated stably in a continuous mode-locked regime throughout the measurement period. This confirms the long-term stability of the mode-locking over extended operation. However, a decrease in stability is expected for longer timescales due to low-frequency noise present in a lab environment and can be addressed through careful packaging and more robust housing.

**Fig. 5:**
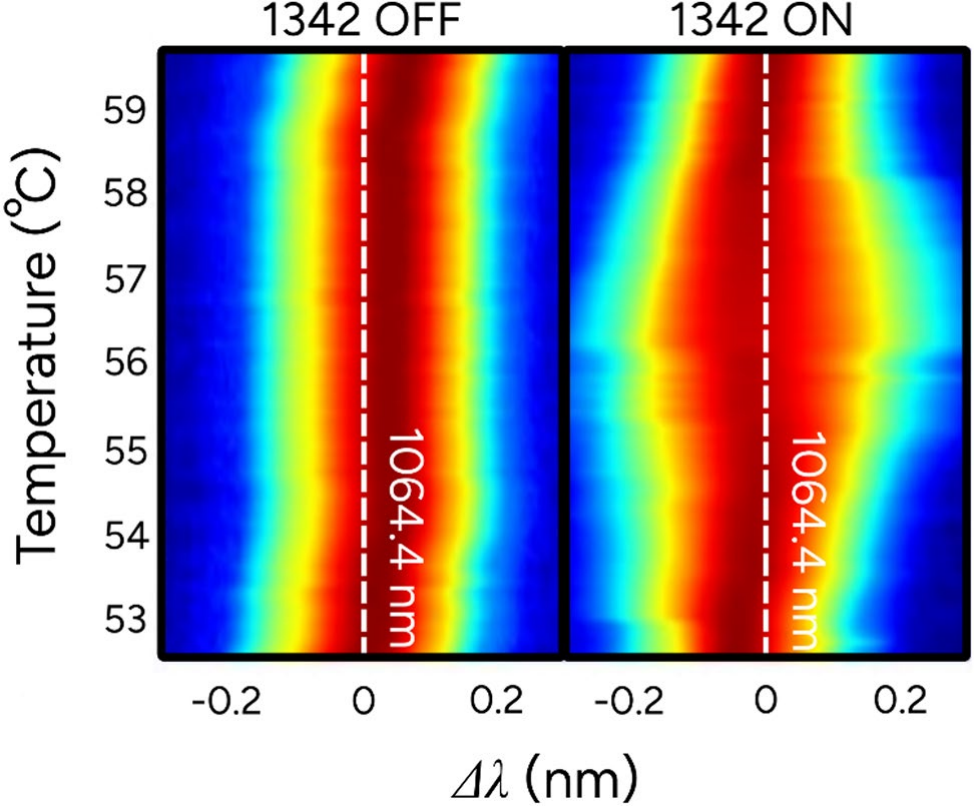
Spectrum is measured using an OSA for different temperatures of the KTP crystal, where $\:{\Delta\:}\lambda\:$ shows the deviation from the center wavelength line at 1064.4 nm. Two cases are shown, one where the 1342 nm arm is turned off and one when it is on.

To further study the underlying mechanism of the mode-locking regime, the spectrum of the 1064 nm laser was measured while tuning the temperature of the nonlinear crystal. Figure 5 shows the bandwidth of the output using an optical spectrum analyzer with a resolution of 70 pm. The results are recorded for two cases; one where the 1342 nm laser was turned on and the other where it was turned off. The spectrum only exhibits broadening for the case when the 1342 nm is active. The broadening occurs between 53 °C and 59 °C; however, stable short pulse mode-locking only occurs between 55 °C and 58 °C. The peak broadening corresponds to ~ 56 °C, which also correlates with the temperature region with the shortest and most stable pulse train. For temperatures outside of this range, the SFM-related loss modulation is less efficient resulting in longer pulse generation by XAM. The peak intensity is then not sufficient for spectral broadening and pulse compression to take place. Stable mode-locking therefore requires precise temperature control of the nonlinear crystal, which is achieved in our setup using a Peltier-controlled crystal mount and an enclosed laser housing. The CW spectrum has a bandwidth of ~ 80 pm (21.2 GHz), and for the peak broadening for pulsed operation a bandwidth of 150 pm (39.7 GHz) is achieved.

**Fig. 6:**
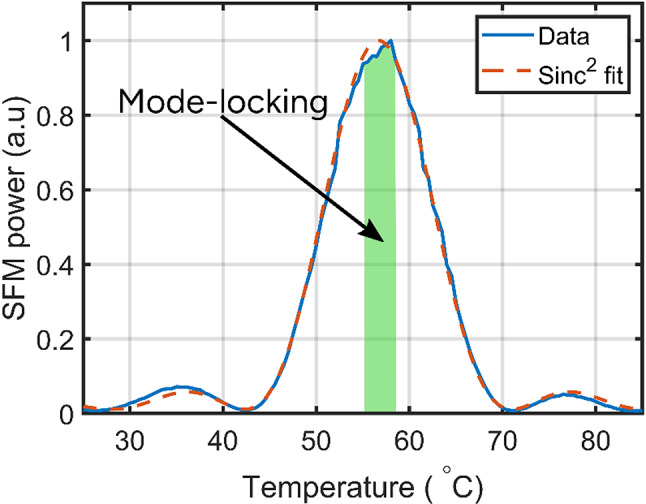
SFM phase matching curve of the intra-cavity KTP crystal used in this study. The green region indicates the temperature range where cascaded mode-locking successfully self-started.

A PPKTP crystal with the same grating period and two independent 1064 nm and 1342 nm lasers were used to measure the SFM phase matching curve (see Fig. 6). The temperature range where the spectral broadening and pulse compression at 1064 nm are observed is marked with the green area in Fig. 6. This demonstrates that mode-locking is sensitive to the phase mismatch for the SFM process and requires high SFM efficiency to drive the dual cavity system into a bright-dark synchronized pulse train generation. As shown in our previous results^18^, the long pulse regime was self-established when the phase mismatch, $\:{\Delta\:}k$, for SFM was near zero. Then the synchronized bright-dark pulse oscillation, i.e. the state where SFM is maximally suppressed becomes the state with lowest losses. This synchronized operation corresponds to a passively phase-locked state between the two cavities. The nonlinear phase-matched SFM interaction introduces intracavity losses that are minimized only when the bright and dark pulse trains in both cavities are temporally coincident, and their spectral phases are locked in such a way that the SFM process efficiency is minimized. Any deviation from this synchronized condition increases SFM efficiency and thus the loss, effectively penalizing out-of-phase operation. If the roundtrip times are matched and the nonlinear interaction is sufficiently efficient, this regime is self-starting and self-maintaining. This nonlinear loss mechanism acts as a passive feedback process that stabilizes the relative phase between the cavities. That is the essence of XAM.

**Fig. 7:**
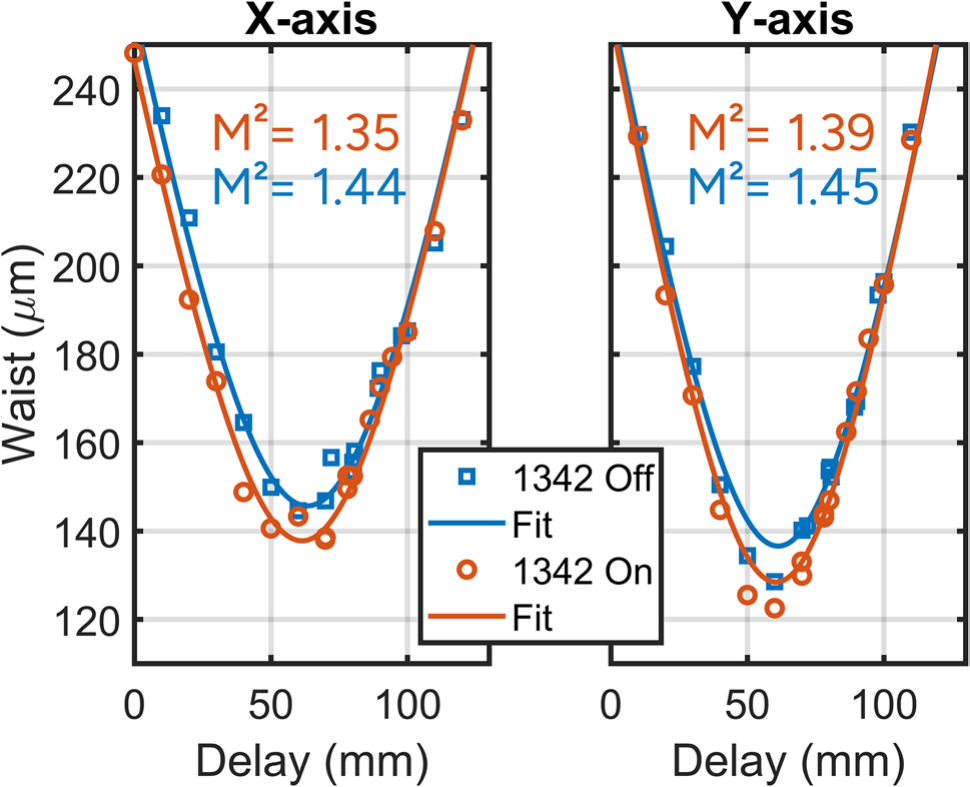
M^2^ measurement in x-axis (horizontal) and y-axis (vertical) with the 1342 nm turned on or off. The M^2^ measurement indicates a tighter spatial mode when the 1342 nm cavity is active.

An important observation is the transverse mode alteration induced by the 1342 nm laser on the 1064 nm beam seen in Fig. 7. This was quantified using a commercial CMOS-based beam profiler under two conditions: with the 1342 nm laser on and off. The results demonstrate that when the 1342 nm laser is active, it forces the 1064 nm mode profile closer to the fundamental mode. Specifically, the M² value on the x-axis (horizontal) decreases from 1.44 to 1.35 and on the y-axis (vertical) from 1.45 to 1.39. The change in 1064 nm mode profile depended on the 1342 nm power and was present for pump powers under threshold for mode-locking. This indicates that the mode alteration was due to SFM loss minimization and was essential for reaching the CSM short pulse generation regime. As a result, the beam area within the PPKTP crystal decreases by 14% when the 1342 nm laser is active compared to when it is turned off.

In the dual-cavity 1064 nm and 1342 nm laser, a short pulse generation regime can only be realized for the configuration where the 1064 nm cavity was generating a bright pulse train, and the 1342 nm cavity was producing synchronized dark pulses. As shown in^18^, it could be arranged that the 1342 nm cavity was producing bright pulses. However, compression could not be achieved in a normal-dispersion cavity for such a configuration because the effective cascaded Kerr coefficient for 1342 nm is positive. Transitioning from long-pulse, XAM-dominated regime required an increase in the 1064 nm circulating peak power and careful positioning of the PPKTP crystal in the intra-cavity focus to take advantage of the Kerr lensing effect that promotes lower-loss spatial mode.

To better understand the dynamics of the pulse compression observed in this regime, the effective nonlinear refractive index generated by the cascaded $\:{\chi\:}^{\left(2\right)}:{\chi\:}^{\left(2\right)}$ interaction is calculated. The cascaded second-order nonlinearity, facilitated by the phase mismatch $\:{\Delta\:}k$ in the KTP crystal for the SHG of 1064 nm, plays a crucial role in dictating the behavior of the pulse compression mechanism. By quantifying the contribution of both the native Kerr nonlinearity and the effective Kerr coefficient introduced by the cascaded nonlinearity, we can evaluate whether the system operates in the soliton compression regime. Specifically, the soliton number, *N*, which governs the nature of pulse compression, can be determined by examining the characteristic nonlinear and dispersive lengths in the cavity^19^. The phase mismatch is given by:

1 $$\:\varDelta\:k={k}_{2\omega\:}-2{k}_{\omega\:}-2\pi\:/\varLambda\:\:\:\:\:\:\:\:\:\:\:\:\:\:\:\:\:\:\:\:\:\:\:\:\:\:\:\:\:\:\:\:\:\:\:\:\:\:\:\:\:\:\:\:\:\:\:\:\:\:\:\:\:\:\:\:\:\:$$

Where $\:{k}_{2\omega\:}$ and $\:{k}_{\omega\:}$ are the wavenumbers for the second harmonic (SH) and fundamental wave (FW), respectively. The third term corresponds to the momentum imposed by the periodic structure with the periodicity Λ. The phase mismatch can be calculated for 1064 nm to $\:{\Delta\:}k=\:+0.21\mu\:{\text{m}}^{-1}$. The effective Kerr nonlinearity at 1064 nm can be calculated as^9,16^

2 $$\:{n}_{2}^{casc}=\frac{2\omega\:{d}_{33}^{2}}{{n}_{\omega\:}^{2}{n}_{2\omega\:}{c}^{2}{\epsilon}_{0}\varDelta\:k}\:\left(sinc\left(\varDelta\:kL\right)-1\right)\:\:\:\:\:\:\:\:\:\:\:\:\:\:\:\:\:\:\:\:\:\:\:\:\:\:\:\:\:\:\:\:\:\:\:\:\:\:\:\:\:\:\:\:\:$$

Where$\:\:{n}_{2\omega\:}$ and $\:{n}_{\omega\:}$ represent the refractive indices for the SH and FW, respectively, and *L* is the propagation length. The nonlinear coefficient, $\:{d}_{33}$, for an input polarization parallel to the c-axis of KTP is 16.9 pm/V^20^. Using this, the effective Kerr coefficient for 1064 nm was calculated to be $\:{n}_{2}^{casc}$ = $\:-3.3\times\:{10}^{-19}{\text{m}}^{2}/\text{W}$. When combined with the native Kerr coefficient, $\:{n}_{2}^{nat}=2.4\times\:{10}^{-19}\:{\text{m}}^{2}/\text{W}\:\left[4\right]$, the total effective Kerr coefficient becomes $\:{n}_{2}^{I}={n}_{2}^{casc}+\:{n}_{2}^{nat}=\:-0.9\times\:\:{10}^{-19}{\text{m}}^{2}/\text{W}$. This indicates that the total Kerr effect will act as a negative lensing element within the cavity. Furthermore, the Kerr coefficient related to the electric field is calculated as $\:{n}_{2}=\frac{1}{2}{n}_{2}^{I}cn{\epsilon}_{0}=\:-2.19\times\:{10}^{-19}{\text{m}}^{2}/{\text{V}}^{2}$.

Before the soliton number, *N*, can be calculated, the characteristic nonlinear and dispersive lengths must first be estimated. The roundtrip dispersion in the cavity is mainly due to the contributions of dispersion in the laser crystal, estimated at 173 $\:\text{f}{\text{s}}^{2}/\text{m}\text{m}$, and in the PPKTP crystal, which is 194 $\:\text{f}{\text{s}}^{2}/\text{m}\text{m}$, yielding a total roundtrip dispersion of $\:{\beta\:}_{2}=734\:\text{f}{\text{s}}^{2}/\text{m}\text{m}$. With this, the dispersive length is

3 $$\:{L}_{d}=\frac{{\tau\:}_{p}^{2}}{\left|{\beta\:}_{2}\right|}=267\:m\:\:\:\:\:\:\:\:\:\:\:\:\:\:\:\:\:\:\:\:\:\:\:\:\:\:\:\:\:\:\:\:\:\:\:\:\:\:\:\:\:\:\:\:\:\:\:\:\:\:\:\:\:\:\:\:\:\:\:\:\:\:\:\:$$

Next, the nonlinear length, $\:{L}_{nl}$, must be determined. It represents the distance over which nonlinear effects, driven by the peak intensity of the pulse, accumulate and affect the pulse dynamics. In our setup, the beam waist inside the KTP crystal is 75 μm, leading to an intra-cavity peak intensity of approximately 490 GW/m^2^. The nonlinear length then becomes:

4 $$\:{L}_{nl}=\frac{{n}_{\omega\:}^{2}{\epsilon}_{0}c}{2\left|{n}_{2}\right|kI}=3.80\:m\:\:\:\:\:\:\:\:\:\:\:\:\:\:\:\:\:\:\:\:\:\:\:\:\:\:\:\:\:\:\:\:\:\:\:\:\:\:\:\:\:\:\:\:\:\:\:\:\:\:\:\:\:\:\:\:\:\:\:$$

With both the dispersive length and the nonlinear length calculated, the soliton number *N* can now be determined. The soliton number is calculated using the square root of the ratio of the dispersive length to the nonlinear length:

5 $$\:N=\sqrt{\frac{{L}_{d}}{{L}_{nl}}}\approx\:8.4\:\:\:\:\:\:\:\:\:\:\:\:\:\:\:\:\:\:\:\:\:\:\:\:\:\:\:\:\:\:\:\:\:\:\:\:\:\:\:\:\:\:\:\:\:\:\:\:\:\:\:\:\:\:\:\:\:\:\:\:\:\:\:\:\:\:\:\:\:\:\:\:$$

The calculated soliton number of approximately 8.4, well above unity, confirms that the system operates in the soliton compression regime, where nonlinear effects dominate the pulse dynamics and enable substantial pulse shortening. Lastly, the extent of spectral broadening due self-phase modulation (SPM) can be estimated. The spectral broadening per roundtrip can be calculated using^19^:

6 $$\:df=\frac{1.72{L}_{qpm}}{{\tau\:}_{p}{L}_{nl}}\approx\:298\:MHz\:\:\:\:\:\:\:\:\:\:\:\:\:\:\:\:\:\:\:\:\:\:\:\:\:\:\:\:\:\:\:\:\:\:\:\:\:\:\:\:\:\:\:\:\:\:\:\:\:\:\:\:\:\:\:$$

The grating length in the PPKTP crystal is denoted as $\:{L}_{qpm}$. To estimate the accumulated SPM broadening, the cavity photon lifetime must first be determined, as this will indicate how many roundtrips are required to reach the steady state in the cavity and thus will allow us to estimate the cumulative spectral broadening due to SPM. The cavity lifetime is influenced by the losses experienced within the cavity. The total logarithmic loss γ is calculated to be approximately 0.077. The cavity photon lifetime $\:{\tau\:}_{c}$ is then determined by:

7 $$\:{\tau\:}_{c}=\frac{1}{{f}_{rep}\gamma\:}\approx\:468\:ns\:\:\:\:\:\:\:\:\:\:\:\:\:\:\:\:\:\:\:\:\:\:\:\:\:\:\:\:\:\:\:\:\:\:\:\:\:\:\:\:\:\:\:\:\:\:\:\:\:\:\:\:\:\:\:\:\:\:\:\:\:$$

where $\:{f}_{rep}\:$is the repetition rate of 275 MHz. The number of roundtrips within this cavity lifetime is given by:

8 $$\:{N}_{rountrips}=\:{\tau\:}_{c}{f}_{rep}\approx\:129\:\:\:\:\:\:\:\:\:\:\:\:\:\:\:\:\:\:\:\:\:\:\:\:\:\:\:\:\:\:\:\:\:\:\:\:\:\:\:\:\:\:\:\:\:\:\:\:\:\:\:\:\:\:$$

Finally, the accumulated SPM broadening is found by multiplying the number of roundtrips by the SPM broadening per roundtrip, yielding a total broadening of approximately 38.4 GHz. This result is in good agreement with the experimentally found bandwidth of 39.7 GHz. This also highlights the limit to the compression, where previous works have demonstrated shorter pulse durations using CSM, in this work we are limited by the photon lifetime and the SPM–related spectral broadening. The effect of the negative Kerr lens due to cascaded second-order interaction is relatively small in this picosecond pulse regime, but not negligible. In fact, it is crucial in promoting a short pulse oscillation regime in the spatial mode with lower losses.

## Conclusion

In this work, we have successfully demonstrated significant pulse compression in the picosecond regime by using CSM in combination with XAM in a dual-cavity setup. The results from previous research We show that the system can operate in the soliton compression regime with stable picosecond pulses at a high repetition rate of 275 MHz. The results highlight the capability of CSM to generate near-transform limited pulses as short as 14 ps. Output power scaling should be achievable by increasing pump power, increasing output coupler transmission, and maintaining sufficient intra-cavity peak intensity for SPM to broaden the spectrum over the cavity lifetime.

A key aspect of the system is the interaction between the synchronized bright and dark pulse trains. Ideally, only the bright pulse experiences the Kerr effect, as the 1342 nm laser effectively disappears and creates an open temporal window for soliton compression of the 1064 nm bright pulse. The SFM process facilitates XAM leading to bright-dark operation, while the cascaded SHG and back-conversion induce negative effective Kerr nonlinearity required for a very substantial increased pulse compression for the bright pulse under normal dispersion conditions.

## Data Availability

Data Availability Statement (DAS). Data underlying the results presented in this paper are not publicly available at this time but may be obtained from brunze@kth.se upon reasonable request.
